# Buoyant force-induced continuous floating and sinking of Janus micromotors†

**DOI:** 10.1039/c8ra05844j

**Published:** 2018-09-26

**Authors:** Meisheng Wu, Yuki Koizumi, Hiroki Nishiyama, Ikuyoshi Tomita, Shinsuke Inagi

**Affiliations:** Department of Chemical Science and Engineering, School of Materials and Chemical Technology, Tokyo Institute of Technology 4259 Nagatsuta-cho, Midori-ku Yokohama 226-8502 Japan inagi@echem.titech.ac.jp wumeisheng@njau.edu.cn; Department of Chemistry, College of Sciences, Nanjing Agricultural University 1 Weigang Nanjing 210095 P. R. China

## Abstract

A novel bubble-induced ultrafast floating and sinking of micromotors based on the difference between buoyant force and gravity is proposed. Asymmetric micromotors were prepared by modification with Au and Pt layers for the two faces of glassy carbon beads (GCBs) by the bipolar electrodeposition technique. After the accumulation of enough oxygen bubbles by the decomposition of H_2_O_2_ at the Pt layer, the upward net force acting on the micromotor drove its movement to the air/solution interface. In order to reverse the direction of net force for the sinking of the micromotors, sodium dodecyl sulfate (SDS) was added into the fuel solution, which could facilitate the release of bubbles and decrease the diameter of the bubbles. However, the lifetime of the bubbles was increased significantly. After the addition of a small amount of salt, the lifetime of the bubbles was obviously reduced. As a consequence, the breakup of bubbles on the micromotor changed the direction of the net force from up to down which pulled the micromotor down to the bottom of the solution. The velocity of the micromotor was dependent on the net force exerted on the micromotor, leading to an ultrafast motion of the micromotor. It still reached 1.2 cm s^−1^ after 3 h. Moreover, the simple asymmetric deposition technique showed great promise for the further application of the micromotors in bioanalysis and environmental remediation.

## Abstract 2

Novel bubble-induced ultrafast moving of micromotors based on the difference between buoyant force and gravity is proposed.

## Graphical abstract

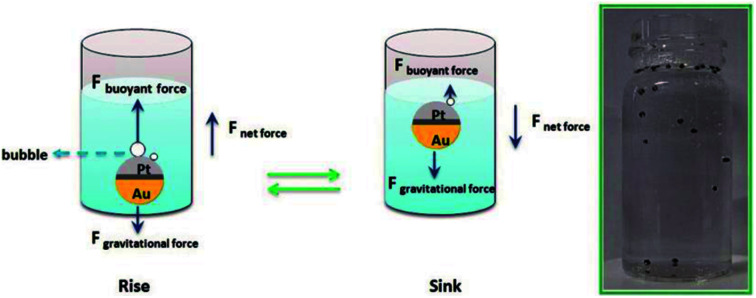

## Introduction

Micro/nanomotors which can perform diverse tasks including target isolation,^1,2^ environmental remediation,^3–5^ repair cracks,^6^ and biosensing^7–9^ have attracted increasing attention. These miniaturized objects move in a fuel solution based on various types of mechanisms, such as bubble propulsion,^10–12^ self-electrophoresis,^13,14^ and self-diffusiophoresis.^15,16^ Among these driving forces, bubble propulsion is an effective approach for the locomotion of large objects due to the strong momentum produced by the detachment of bubbles from a catalytic layer. Therefore, it can achieve higher velocity than the other two mechanisms.

Up to now, many approaches have been developed to fabricate bubble-propelled micromotors, including sputter coating,^11^ template-assisted electrodeposition,^17^ and rolled-up nanotechnology.^3^ The latter two approaches are used to fabricate tubular microjets which have higher velocity than Janus microspheres prepared by sputtering. For example, rolled-up micromotor can reach ultrafast speed of 10 mm s^−1^ ^18^ due to the fast bubble formation^19^ and detachment frequency^20^ from concave than convex and flat surface, while the speed of spherical Janus particles is about several hundreds of μm s^−1^.^10^ Besides, the velocity of a tubular jet can be improved by increasing its length, which enables the application of micromotors in a low concentration of hydrogen peroxide (H_2_O_2_) solution.^21^ All of these make Janus microspheres less efficient as bubble-propelled micromotors which limit their applications.

To better control the speed of micromotors, it is necessary to prepare micromotors with controllable surface area of a catalyst layer. Bipolar electrochemistry, as a powerful technique, has attracted great interest in the fabrication of Janus particles with tunable surface coverage of particles by applying various external voltages on driving electrodes.^22–25^ Conductive particles are placed in the electrolyte solution to serve as bipolar electrodes. When the voltage is sufficiently high, redox reactions can take place at the two faces of all of these conductive particles simultaneously. Since there is no direct electric connection between the bipolar electrodes and external power source, plenty of Janus micromotors can be obtained at the same time.

In this work, we focus on proposing a novel locomotion mechanism on the basis of buoyant force of bubbles for floating and sinking of Janus micromotors in order to achieve ultrafast movement. Janus micromotor was prepared by bipolar deposition of Au and Pt at the two faces of glassy carbon beads (Au–GCB–Pt), respectively. After the generation of enough amounts of large bubbles on the micromotor, the upward buoyant force would overcome the downward gravity and drove its motion to air/solution interface. Sodium dodecyl sulfate (SDS) was added to reduce the surface tension of solution and accelerate the release of bubbles. However, the lifetime of bubbles was increased accordingly. All of micromotors at solution surface were surrounded by plenty of oxygen bubbles which impeded the sinking of them. Previous study has indicated that the addition of small amount of salt can decrease the lifetime of bubbles because of the reduced electrostatic forces and surface tension.^26^ Therefore, low concentration of sodium sulfate (Na_2_SO_4_) was added into the mixture solution of H_2_O_2_ and SDS. As expected, the amount and lifetime of bubbles at solution surface were decreased obviously. After the bursting or release of bubbles, the direction of the net force changed due to the decrement of buoyant force, which pulled the micromotor down to the bottom of solution. The formation and breakup of bubbles on Au–GCB–Pt enabled it to move up and down with ultrafast speed of 1.5 cm s^−1^ in low concentration of H_2_O_2_ (0.526%) for several hours which was much higher than that in previous work.

## Results and discussion

### Bipolar deposition of Janus GCB

**Scheme 1:**
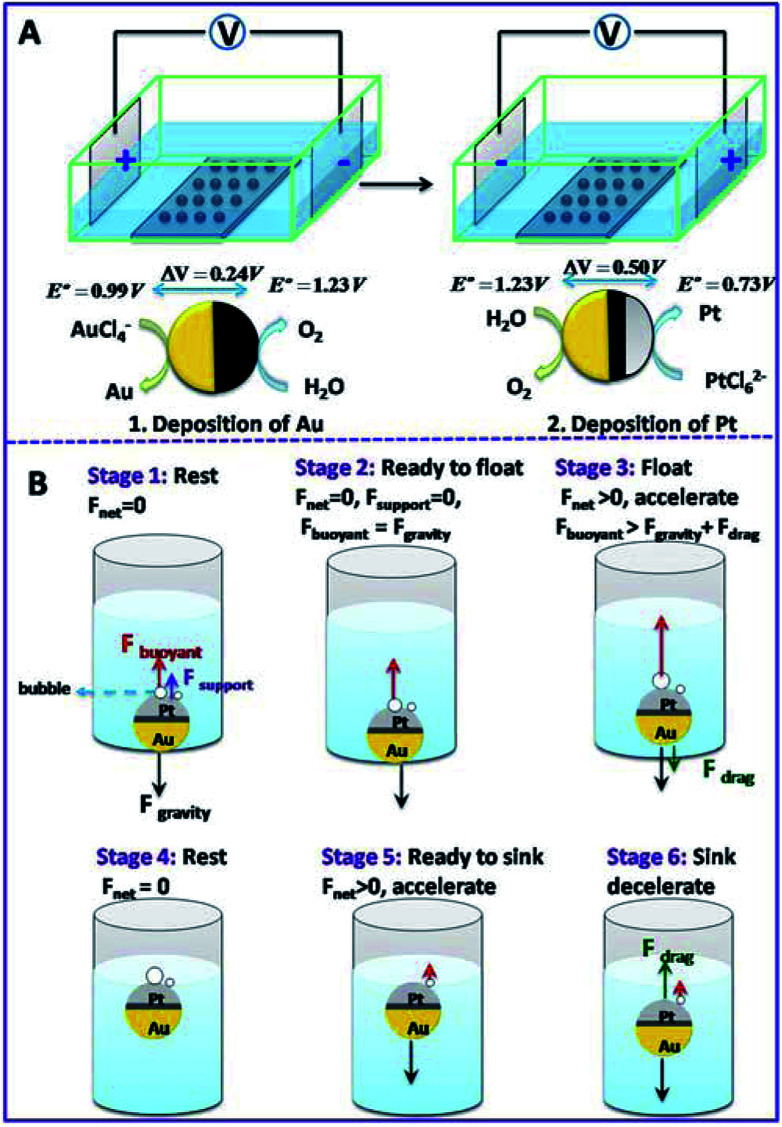
(A) Schematic illustration of the fabrication process of Au–GCB–Pt Janus particles. (B) Mechanism of bubble induced floating and sinking of a micromotor.

Bipolar electrochemistry has been widely used for the fabrication of Janus particles with controllable surface coverage by applying various voltages. Here, it was used to deposit Au and Pt nanoparticles (NPs) at the two faces of a GCB, respectively. Pt NPs serve as catalyst to decompose H_2_O_2_ and produce oxygen bubbles which generate enough buoyant force for the motion of the GCB. Au film is used to form asymmetric structure which can conjugate functional groups for the further application of the micromotors. Scheme 1A shows the setup of bipolar deposition. AuCl_4_^−^ was reduced at the cathode of GCBs to form Au NPs and water was oxidized at the anode of GCBs when the electric field was sufficiently high. Then the electric field was reversed to deposit Pt NPs at the opposite face of Au. The morphologies of Janus particles were characterized with SEM. The average surface coverage of Au reached 49.6% when the voltage was 50 V (Fig. S1A†). Since the decomposition of H_2_O_2_ depended strongly on the area of Pt layer, we prepared Au–GCB–Pt with different surface coverages of Pt. As can be seen from Fig. S1B to 1F,† the surface coverage of Pt film increased obviously with the increase of deposition voltage from 30 to 70 V. It could reach 43.2% when the voltage was 70 V.

### Locomotion of Au–GCB–Pt in fuel solution

The moving object in solution experiences three forces: upward buoyant force (*F*_buoyant_), downward gravitational force (*F*_gravity_), and drag force (*F*_drag_, opposite to the direction of velocity) caused by the viscosity of solution. Net force acted on the motor is given by eqn (1) and (2).

1 *F*_net_ = *F*_gravity_ − *F*_buoyant _ − *F*_drag_Sink

2 *F*_net_ = *F*_buoyant_ − *F*_gravity_ − *F*_drag_Float

When Au–GCB–Pt is immersed in fuel solution, the decomposition of H_2_O_2_ produces a large amount of oxygen bubbles which generates extra buoyant force on Au–GCB–Pt (*F*^bubble^_buoyant_). Therefore, the total buoyant force (*F*_buoyant_) equals the weight of solution displaced by Au–GCB–Pt (*F*^GCB^_buoyant_) and bubbles on Au–GCB–Pt surface (*F*^bubble^_buoyant_).

3 *F*_buoyant_ = *F*^bubble^_buoyant_ + *F*^GCB^_buoyant_ = *ρ*_solution_*g*∑*V*_bubble_ + *ρ*_solution_*gV*_GCB_

4
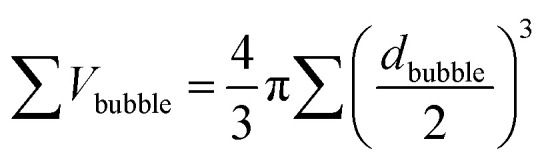
*ρ*_solution_ is the density of fuel solution, *g* is the gravity acceleration (9.8 m s^−2^), ∑*V*_bubble_ is the sum of volumes of the bubbles on GCB surface, *d*_bubble_ is the diameter of bubble.

The magnitude of drag force can be calculated by using Stokes's law:^27^

5 *F*_drag_ = 6π*ηγυ*

*η* denotes the viscosity of solution, *γ* is the radius of moving object, *υ* is the speed of object.

For Au–GCB–Pt resting on the bottom of container (*F*_drag_ = 0, stage 1 in Scheme 1B), the downward gravity is balanced by the upward buoyant force and supporting force (*F*_support_) exerted by the container. With the generation and growth of bubbles on Au–GCB–Pt, *F*_buoyant_ is increasing and *F*_support_ is decreasing in order to maintain the balance of Au–GCB–Pt. When *F*_buoyant_ = *F*_gravity_, *F*_support_ is equal to zero and Au–GCB–Pt is ready to float. With the improvement of buoyant force, Au–GCB–Pt moves upward in fuel solution (stage 3 in Scheme 1B). Meanwhile, *F*_drag_ is exerted on the moving object in the same direction as gravity. Due to the low density of bubbles adhered on Au–GCB–Pt, Au–GCB–Pt will adjust its direction autonomously with the Pt layer oriented upward during the floating process. Then Au–GCB–Pt arrives at solution surface and the velocity of it in vertical direction is zero (stage 4 in Scheme 1B) before dropping into solution. After the breakup and detachment of bubbles, the net force changes to downward, leading to the dropping of Au–GCB–Pt into solution (stage 5 in Scheme 1B). With the further formation of bubbles on Au–GCB–Pt, it floats to the solution surface again. As a result, this new type of locomotion mechanism enables the continuous floating and sinking of micromotors in a fuel solution.

**Fig. 1:**
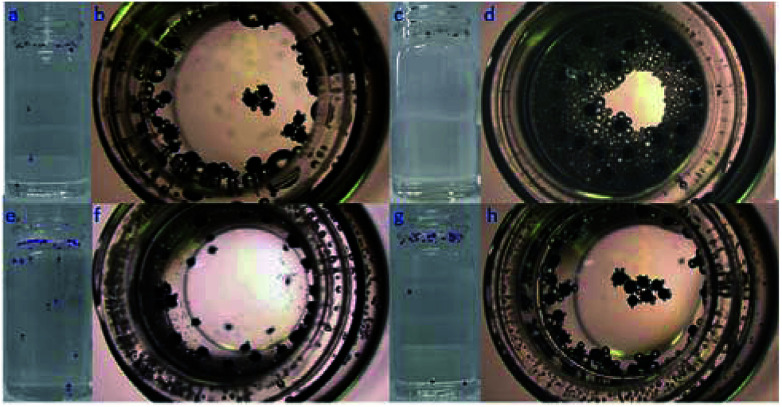
Side view (a, c, e and g) and top view (b, d, f and h) of Au–GCB–Pt in various fuel solutions. (a and b) 0.526% H_2_O_2_ solution. (c and d) Mixture solution of 0.526% H_2_O_2_ and 0.021 g L^−1^ SDS. (e and f) Mixture solution of 0.526% H_2_O_2_, 0.021 g L^−1^ SDS, and 0.185 M Na_2_SO_4_. (g and h) Mixture solution of 0.526% H_2_O_2_ and 0.185 M Na_2_SO_4_. Images in (a, c, e, and h) were extracted from movie and obtained by overlapping three consecutive frames. The color of micromotors in these three frames was changed to black, red, and blue, respectively. Time interval was 0.033 s. All of these images were recorded after mixing Au–GCB–Pt with fuel solution for 10 min except (a) (2 min). The volume of fuel solution was 6.0 mL.

In order to prove the proposed motion mechanism, Au–GCB–Pt was submerged into 0.526% H_2_O_2_ solution. Three consecutive frames were extracted from movie and overlapped. GCBs in these images were changed to black, red, and blue with ImageJ to illustrate the motion direction. Small amount of Janus particles could rise and sink in this solution in the first 2 min (Fig. 1a, Movie S1†). However, after 10 min (Movie S1†), all of the micromotors located at the solution surface. It was caused by the high buoyant force produced by the large amount of big bubbles attached on Au–GCB–Pt which impeded the sinking of the micromotors (Fig. 1b). In order to solve the problem, anionic surfactant, sodium dodecyl sulfate (SDS), was added into the fuel solution to facilitate the release of bubbles due to the electrostatic interaction between the positively charged Pt surface during the decomposition of H_2_O_2_.^28,29^ As expected, the bubble releasing rate was improved significantly (Fig. 1c and d). However, it also increased the amount of bubbles and their lifetime greatly (Movie S1†). As a result, all of the micromotors were surrounded by plenty of bubbles and no micromotor could fall into solution.

Then we tried to decrease the rest time of bubbles by adding low concentration of salt.^26,30^ Movie S1† clearly demonstrated the continuous floating and sinking of Au–GCB–Pt in the mixture solution of H_2_O_2_, SDS, and low concentration of Na_2_SO_4_. Fig. 1e indicated that six micromotors were moving in this fuel solution and two of them were dropping. The amount of bubbles at solution surface reduced apparently after the addition of Na_2_SO_4_ (Fig. 1f). Movie S2† (×2 frame rate) was captured at high magnification which exhibited that Au–GCB–Pt reoriented with the Pt layer upward due to the low density of bubbles. It confirmed that the floating of Au–GCB–Pt was caused by the increased buoyant force instead of the propulsion of bubbles.

For comparison, we also explored the motion of Au–GCB–Pt in the absence of SDS (Fig. 1g and h). Although the amount of bubbles at solution surface (Fig. 1h) was lower than that in the presence of SDS (Fig. 1f), the diameter of them was too large. Thus, no GCB could sink in this fuel solution (Movie S1†), confirming that Au–GCB–Pt could only move up and down in the mixture solution of H_2_O_2_, SDS, and electrolyte (Na_2_SO_4_).

**Fig. 2:**
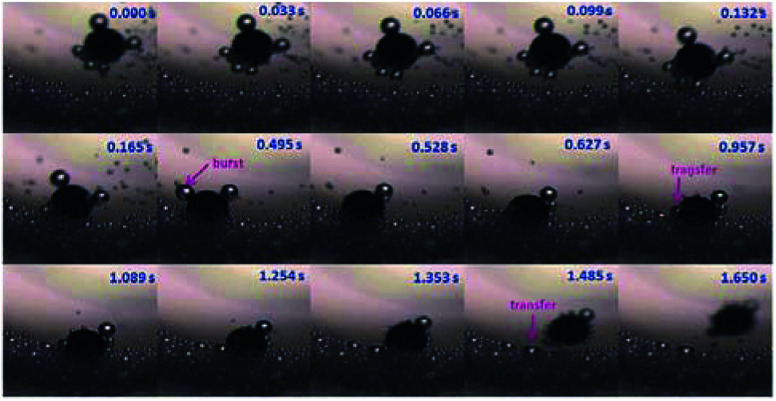
Time lapse images of Au–GCB–Pt were extracted from Movie S3† (1.6× magnification objective lens). 6 mL fuel solution containing 0.526% H_2_O_2_, 0.021 g L^−1^ SDS, and 0.185 M Na_2_SO_4_ was added into a glass vial. The volume of fuel solution was 6.0 mL.

In order to confirm the proposed motion mechanism, time lapse images were taken from Movie S3† and shown in Fig. 2. At 0.495 s, one of large bubbles burst as indicated by red arrow, but Au–GCB–Pt did not fall into solution. After the transfer of the bubble from the micromotor to solution surface (0.957 s), it started sinking process. According to Movie S3,† both of the burst and detachment of bubbles could reduce the buoyant force acted on the micromotor, leading to the sinking of it.

**Fig. 3:**
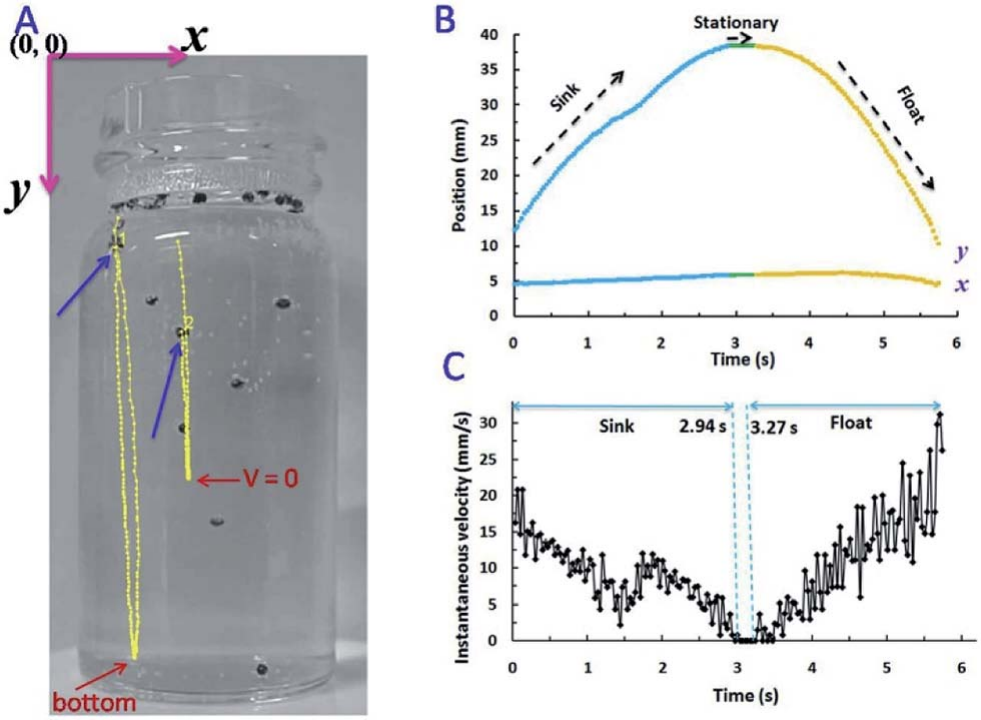
(A) Trajectory of Au–GCB–Pt in fuel solution containing 0.526% H_2_O_2_, 0.021 g L^−1^ SDS, and 0.185 M Na_2_SO_4_. Image was extracted from movie. Coordinates (B) and instantaneous speed (C) of micromotor-1 as a function of time. The volume of fuel solution was 6.0 mL.

The tracking lines of two micromotors and the velocity of motor-1 were displayed in Fig. 3. Blue arrows indicated the original position of them (Fig. 3A). Micromotors dropped from solution surface to the bottom of solution (micromotor-1) or the position where its velocity was reduced to zero (micromotor-2). After the accumulation of enough buoyant force, it began moving upward. Coordinates of micromotor-1 as a function of time were displayed in Fig. 3B. *x* coordinate had no obvious change during the sinking and floating processes.

*y* coordinate decreased from 0 to 2.94 s during the sinking process. The instantaneous velocity was presented in Fig. 3C. Highest velocity could be observed when Au–GCB–Pt dropped from solution surface and then the velocity was reduced gradually. It should be noted that the direction of net force during sinking was more complicated than floating. This is because velocity, *F*_net_, and *F*_drag_ were zero when Au–GCB–Pt was at the solution surface (stage 4 in Scheme 1B). If the balance of force was lost at the moment of bubble breakup, Au–GCB–Pt dropped in the solution at an accelerated speed due to the same direction of net force and motion (stage 5 in Scheme 1B). However, the huge drag force (opposite to the direction of motion) caused by the ultra-fast movement of Au–GCB–Pt may impede the sinking of it. According to Newton's laws of force and motion, the net force is in the opposite direction as motion if an object is decelerated.^31,32^ Therefore, the deceleration of Au–GCB–Pt in sinking process (Fig. 3C) implied that the direction of net force changed from downward to upward due to the large drag force and further generation and growth of bubbles on Pt cap (stage 6 in Scheme 1B).

If the velocity of motor reduced to zero before reaching the bottom of container, its motion direction would change to upward, leading to the re-floating of Au–GCB–Pt (see motor 2 in Fig. 3A). Otherwise, it arrived at the bottom of container until enough buoyant force was produced (Fig. 3C, 2.94 to 3.27 s). Then it floated at an accelerated velocity (Fig. 3C, after 3.27 s) and a decreased *y* coordinate was observed, indicating that the net force was upward, even though *F*_drag_ was increasing (eqn (5)) in the opposite direction of moving. As a result, highest instantaneous speed could be obtained at the initial stage of sinking or the final stage of floating process.

**Fig. 4:**
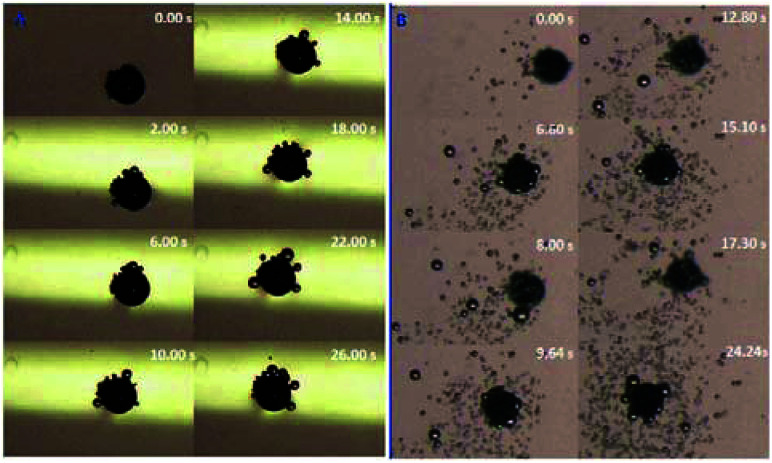
Time lapse images of Au–GCB–Pt placed at solution surface (A) and inside (B) fuel solution in Petri dish. Fuel solution contains 0.526% H_2_O_2_, 0.021 g L^−1^ SDS, and 0.185 M Na_2_SO_4_. Images were extracted from Movie S4.† The volume of fuel solution was 11.4 mL.

In order to further investigate the motion mechanism, Au–GCB–Pt (Movie S4†) was placed at solution surface and submerged in solution, respectively. Fig. 4 shows the time lapse images extracted from Movie S4.† When Au–GCB–Pt was placed on solution surface (Fig. 4A), the hydrophobicity and the bubbles produced on its surface made it partially submerged into solution. It produced an additional upward capillary force for the floating of Au–GCB–Pt. Besides, it could not navigate at solution surface due to the small thrust force produced by the detachment of bubbles in the presence of such a low concentration of H_2_O_2_. If the micromotor was immersed into fuel solution (Fig. 4B), it reached solution surface at 6.60 s. After the breakup of large bubbles, it started moving to the bottom of Petri dish (8.00 s). Then it floated (9.64, 15.1, and 24.24 s) and sunk (12.80 and 17.30 s) in the fuel solution continuously because of the generation and burst (release) of bubbles. All of these results indicate that the sinking of Au–GCB–Pt can only be realized when it is submerged into fuel solution.

In addition, we also studied the effect of deposited particles on the balance of GCB with different surface coverages of Au and Pt. The deposition voltages for Au and Pt were 50 and 20 V, respectively. Half surface of GCB was covered by Au, while no Pt could be seen on the other side (Fig. S2A†) due to the insufficient voltage for the deposition of Pt. Fig. S2B† displayed that all particles located at the bottom of container and no bubble could be seen on Au–GCB–Pt. The bright yellow film (Au) confirmed that Au–GCB–Pt displayed random orientation in fuel solution. It demonstrated that the thickness of Au and Pt have no effect on the balance of motor.

### Optimization of the concentration of fuel solution

Since buoyant force is dependent on the bubble generation and release frequency, the influence of H_2_O_2_ on the average moving number (Fig. S3A†) of micromotors in each frame and velocity (Fig. S3B†) was examined. High concentration of H_2_O_2_ improved the bubble formation rate which produced large buoyant force for the rising of the micromotors. However, it did not lead to the increased average motion number of the micromotors in each frame. This was because the motion number was not only affected by the bubble generation rate, but also controlled by the bubble bursting or releasing rate. The velocity increased when the concentration of H_2_O_2_ increased from 0.263 to 0.526% and then decreased. Therefore, low concentration of H_2_O_2_ was favorable for the buoyant force-induced locomotion.

The motion of micromotors in fuel solutions containing different concentrations of SDS was also investigated (Fig. S4†). The average moving number in each frame increased when the concentration of SDS increased from 0.007 to 0.021 g L^−1^ and then decreased (Fig. S4A†) due to the increased lifetime of bubbles. Fig. S4B† indicated that the average speed showed no obvious change. Thus, 0.021 g L^−1^ of SDS was selected for the following experiments.

The concentration of Na_2_SO_4_ was then optimized and the results are displayed in Fig. S5.† The average motion number increased and reached the highest level when the concentration of Na_2_SO_4_ was 0.123 M and then decreased (Fig. S5A†). Meanwhile, an increased amount of oxygen bubbles could be observed with the concentration of Na_2_SO_4_ (Fig. S6†), which might inhibit the detachment of bubbles. The highest velocity could be obtained when the concentration of Na_2_SO_4_ was 0.185 M (Fig. S5B†).

### Velocity and moving number of micromotors with different surface coverage of Pt

**Fig. 5:**
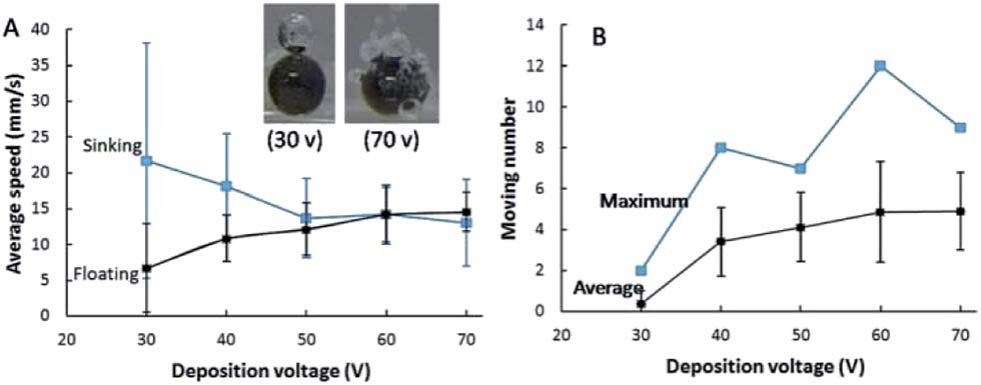
(A) Average floating and sinking speed of Au–GCB–Pt prepared under different deposition voltage of Pt. The speed was calculated by averaging 5 particles. Fuel solution contained 0.526% H_2_O_2_, 0.021 g L^−1^ SDS, and 26.32 g L^−1^ Na_2_SO_4_. Inset was the images of Au–GCB–Pt (deposition voltage of Pt was 30 and 70 V, respectively) in fuel solution. (B) The average and maximum number of Au–GCB–Pt moved in fuel solution were calculated in 600 frames. Deposition voltage of Au was 50 V for 2 min. Deposition time of Pt was 2 min. The volume of fuel solution was 6.0 mL.

Bubble formation rate is enhanced on a micromotor with large surface area of a Pt film, leading to an improved floating rate. Inset in Fig. 5 exhibits that the amount of bubbles generated at micromotors fabricated under deposition voltage of 30 and 70 V. Only one large bubble could be observed on most motors when the deposition voltage of Pt was 30 V due to the small surface area for the decomposition of H_2_O_2_. While plenty bubbles were produced at the micromotor with high deposition voltage of Pt (70 V). It implied that the floating and sinking velocity of micromotors with different surface coverages of Pt might be quite different. The motion of Au–GCB–Pt prepared under different deposition voltages of Pt were presented in Movie S5.†Fig. 5A indicated that a micromotor possessed the lowest floating speed and the highest sinking speed when the deposition voltage of Pt was 30 V. The limited number of bubbles gave rise to low buoyant force, leading to a low upward net force for the rising of the micromotor and a long time for the generation of sufficient buoyant force (about 45 s). After the breakup of the only bubble on the micromotor, the direction of net force reversed and it was nearly equal to the gravity, resulting in a remarkable sinking speed. Importantly, this voltage was insufficient for the deposition of Pt at Au–GCB surface if its diameter was too small. Therefore, no bubbles could be observed on some GCBs surface. All of them led to the lowest number of microspheres moved in the fuel solution (Fig. 5B).

The buoyant force for driving the floating of Au–GCB–Pt could be calculated according to eqn (3) and (4). As shown in Movie S5,† it is difficult to record all bubbles if the catalytic area is too large. But we could calculate *F*^bubble^_buoyant_ when the deposition voltage of Pt was 30 V. Particle started to float until the diameter of bubble on Au–GCB–Pt (760 μm) reached 520 μm. The buoyant force generated by bubble (*F*^bubble^_buoyant_) was approximately 7.2 × 10^−7^ N assuming that the density of fuel solution was 1.0 × 10^3^ kg m^−3^ (density of pure water).

With the increase of deposition voltage, both of bubble generation rate and amount were increased, resulting in an enhanced floating speed and reduced sinking speed due to the large buoyant force. Accordingly, the average and maximum number of micromotors moved in the fuel solution at the same time were enhanced significantly. Both of them reached the highest value when the deposition voltage of Pt was 60 V.

Fig. S7† shows the moving number and velocity of micromotors as a function of time in the mixture solution of H_2_O_2_, SDS, and Na_2_SO_4_ (Movie S6†). After 3 h, the average velocity still reached 1.2 cm s^−1^ which was higher than bubble-propelled micromotors, confirming the excellent swimming performance of heavy micromotors.

### Motion behavior of GCB in fuel solutions containing other electrolytes

**Fig. 6:**
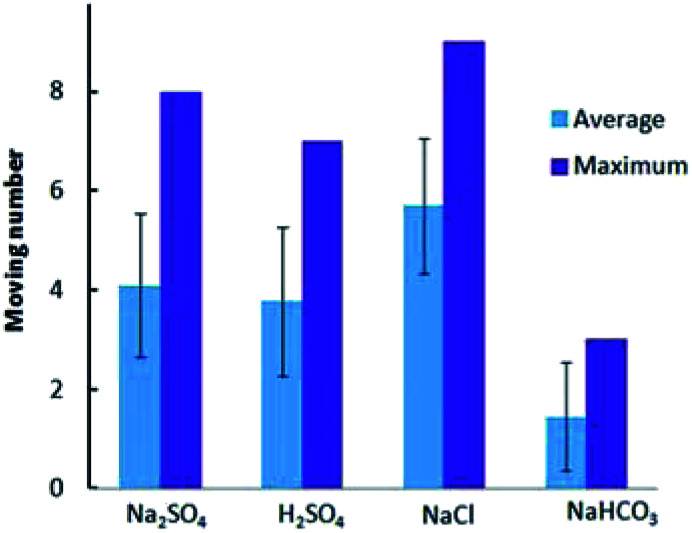
Au–GCB–Pt in fuel solution containing 0.526% H_2_O_2_, 0.021 g L^−1^ SDS and 0.185 M electrolytes. The volume of fuel solution was 6.0 mL.

Then we compared the locomotion of Au–GCB–Pt in fuel solution containing Na_2_SO_4_, H_2_SO_4_, NaCl, and NaHCO_3_, respectively. The continuous swimming of Au–GCB–Pt in fuel solution containing these electrolytes could also be observed (Movie S7†). The average and maximum number of micromotors in these four solutions were shown in Fig. 6. All of these results demonstrated that the addition of low concentration of electrolytes could drive the bubble generation and breakup induced floating and sinking of the micromotors in fuel solution.

## Conclusions

A novel swimming mechanism on the basis of net force (difference between buoyant force and gravitational force) acted on a micromotor was proposed. Buoyant force was the key factor for the floating and sinking of the micromotors in fuel solution which was dependent on the amount and diameter of bubbles adhered on the micromotors. It could be tuned by the concentration of chemicals (H_2_O_2_, surfactant, and electrolyte) and the surface area of catalytic particles. This micromotor possessed ultrafast speed of *ca.* 1.5 cm s^−1^ in low concentration of fuel solution, attributing to the high bubble generation and breakup rate. It broadens the application of micromotors with high density and opens a new horizon for the motion of objects. Moreover, the asymmetric micromotor enables the modification of functional group on the other face of it for the further applications, such as bioanalysis and environmental remediation.

## Experimental

### Reagents and chemicals

Hydrogen tetrachloroaurate (III) tetrahydrate (HAuCl_4_, 99%), hydrogen hexachloroplatinate (IV) hexahydrate (H_2_PtCl_6_·6H_2_O), and hydrogen peroxide (H_2_O_2_, 30%) were purchased from Wako Pure Chemical Industries. Glassy carbon beads (GCBs, diameter of 630–1000 μm, type 2) were obtained from Alfa Aesar. Sodium dodecyl sulfate (SDS) was obtained from Sigma-Aldrich. Sodium bicarbonate (NaHCO_3_), sodium sulfate (Na_2_SO_4_), and sodium chloride (NaCl) were purchased from Kanto Chemical Company. Solutions were prepared using distilled water.

### Apparatus

EC1000SA AC/DC power source (NF Corporation) was used to generate uniform DC electric field for bipolar deposition. Stylus-TG4 camera was used to capture photos and movies at a frame rate of 29.97 fps for 1 min. Movies recorded by this camera were converted to MPG with free HD video converter. Olympus SZX10 stereomicroscope was used to record the movement of micromotors from the top of solution.

VirtualDub software was used to extract segments from movie and converted them into uncompressed videos. ImageJ was used to track and calculate the speed of micromotor. The average moving number of micromotors was calculated in 600 frames. Pixel was converted into physical units by measuring the real width (18.0 mm) and height (40.2 mm) of the glass vial.

### Preparation of Au GCB, Pt GCB, and Au–GCB–Pt

GCBs were rinsed with acetone and water by sonication before bipolar deposition and then dried at 100 °C. After that, GCBs were treated with acid mixture to increase their hydrophilicity. 10 mL of concentrated HNO_3_ (68%) was added into 30 mL of concentrated H_2_SO_4_ (98%) slowly. 1.0 g of GCBs was added into the above acid mixture and sonicated at 50 °C for 2 h. Then GCBs were washed with distilled water for several times until the solution was at neutral pH value and dried at 100 °C.

Grids were created by Adobe Illustrator and then printed on a sheet of A4 paper in order to fabricate GCBs array. The width of lines in grid was 0.5 mm and the center-to-center distance between two parallel neighboring lines was 1.5 mm. The grid paper was cut into small pieces and bonded onto glass substrate (width: 0.5 cm, length: 2.0 cm) with double-sided tape. Then transparent tape was attached on double-sided tape. Finally, 60 GCBs were placed onto it to form a uniform array of 5 × 12 under optical microscope.

Bipolar deposition was conducted in a homemade square electrochemical cell by using two Pt electrodes as driving electrodes (Scheme 1A). The distance between Pt electrodes was 2.5 cm. 3 mL of 1 mM HAuCl_4_ was added into the electrochemical cell and a voltage of 50 V was applied for 2 min. After rinsing with water for several times, 3 mL of 0.5 mM H_2_PtCl_6_ was introduced and a reversed electric filed was applied to deposit Pt nanoparticles (NPs) at the opposite face of GCB. In order to obtain micromotors with various surface areas of Pt, external voltage from 30 to 70 V was applied for 2 min, respectively. The obtained Au–GCB–Pt was rinsed with distilled water and then stored in water at room temperature before use. Oxygen bubbles produced at the anode of GCB due to the oxidation of H_2_O should be removed before and after each deposition experiment.

### Motion of Au–GCB–Pt micromotors in fuel solution

The motion of micromotors in fuel solution was performed by immersing 30 micromotors into 6.0 mL of fuel solution containing 0.526% H_2_O_2_, 0.021 g L^−1^ SDS, and 0.185 M Na_2_SO_4_ in a glass vial. Movies were taken from the side and top of solution.

## Conflicts of interest

There are no conflicts to declare.

## Supplementary Material

RA-008-C8RA05844J-s001

RA-008-C8RA05844J-s002

RA-008-C8RA05844J-s003

RA-008-C8RA05844J-s004

RA-008-C8RA05844J-s005

RA-008-C8RA05844J-s006

RA-008-C8RA05844J-s007

RA-008-C8RA05844J-s008
