# Supplementary material for: Buoyant force-induced continuous floating and sinking of Janus micromotors
Source: RSC Adv. 2018 Sep 26;8(58):33331–7. doi: 10.1039/c8ra05844j (PMC9086439; doi:10.1039/c8ra05844j)
Supplement: RA-008-C8RA05844J-s008 [file RA-008-C8RA05844J-s008.pdf]

Electronic Supplementary Material (ESI) for RSC Advances.
This journal is © The Royal Society of Chemistry 2018

Electronic Supplementary Information

Buoyant Force-Induced Continuous Floating and Sinking of Janus Micromotors

Meisheng Wu[a,b,*], Yuki Koizumi[a], Hiroki Nishiyama[a], Ikuyoshi Tomita[a], Shinsuke Inagi[a,*]

[a]Department of Chemical Science and Engineering, School of Materials and Chemical Technology, Tokyo Institute of Technology, 4259 Nagatsuta-cho, Midori-ku, Yokohama 226-8502, Japan
[b]Department of Chemistry, College of Sciences, Nanjing Agricultural University, 1 Weigang, Nanjing 210095, P.R. China.

Movie S1. Side view of Au-GCB-Pt in 0.526% H_2O_2 solution, mixture solution of 0.526% H_2O_2 and 0.021 g/L SDS, mixture solution of 0.526% H_2O_2, 0.021 g/L SDS and 0.185 M Na_2SO_4, and mixture solution of 0.526% H_2O_2 and 0.185 M Na_2SO_4.

Movie S2. Orientation of Au-GCB-Pt in fuel solution containing 0.526% H_2O_2, 0.021 g/L SDS and 0.185 M Na_2SO_4.

Movie S3. Top view of Au-GCB-Pt in fuel solution containing 0.526% H_2O_2, 0.021 g/L SDS and 0.185 M Na_2SO_4.

Movie S4. Motion performance of Au-GCB-Pt placed on solution surface and in solution. Fuel solution contains 0.526% H_2O_2, 0.021 g/L SDS and 0.185 M Na_2SO_4.

Movie S5. Motion of Au-GCB-Pt with different surface coverage of Pt. Au was deposited at 50 V for 2 min. The deposition voltage for Pt was 30, 40, 50, 60 and 70 V, respectively. Fuel solution contains 0.526% H_2O_2, 0.021 g/L SDS and 0.185 M Na_2SO_4.

Movie S6. Swimming of Au-GCB-Pt in fuel solution containing 0.526% H_2O_2, 0.021 g/L SDS and 0.185 M Na_2SO_4 after 10 min, 1 h, and 3 h.

Movie S7. Swimming of Au-GCB-Pt in fuel solution containing 0.526% H_2O_2, 0.021 g/L SDS and 0.185 M electrolytes (H_2SO_4, NaCl and $NaHCO_3$).

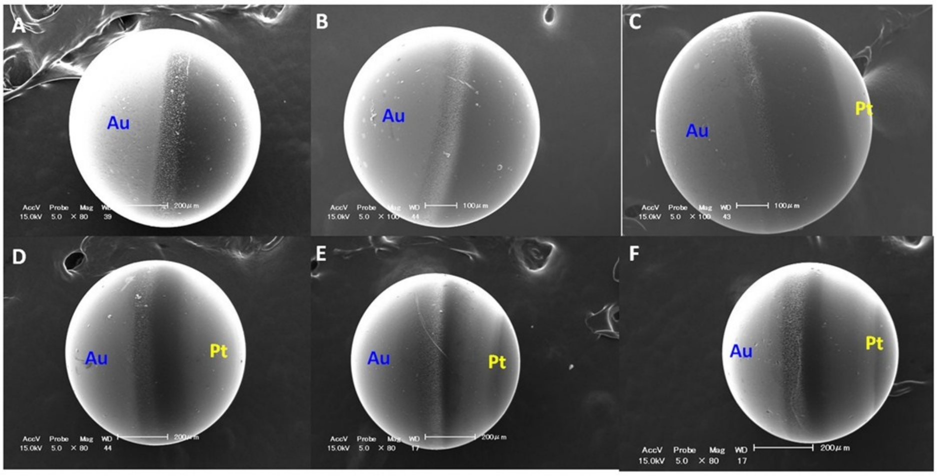

Fig.S1. SEM images of Au-GCB (A) and Au-GCB-Pt (B to F). Au was deposited at 50 V for 2 min. The deposition voltage of Pt NPs from (B) to (F) was 30, 40, 50, 60 and 70 V, respectively. The deposition time of Pt was 2 min.

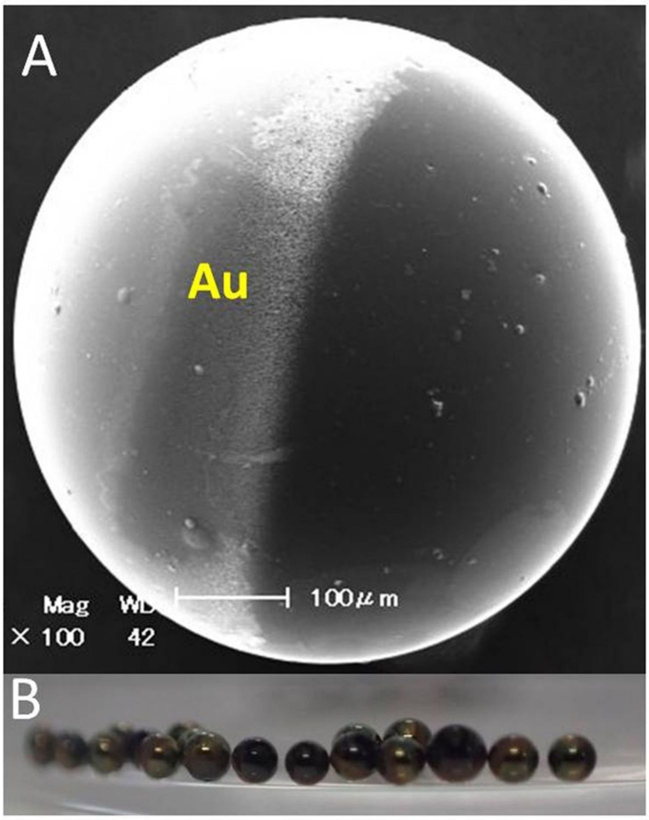

Fig.S2 (A) SEM image of Au-GCB-Pt. (B) Au-GCB-Pt in fuel solution containing 0.526% H_2O_2, 0.021 g/L SDS, and 0.185 M Na_2SO_4. The volume of fuel solution was 6.0 mL. The deposition voltages for Au and Pt were 50 and 20 V, respectively.

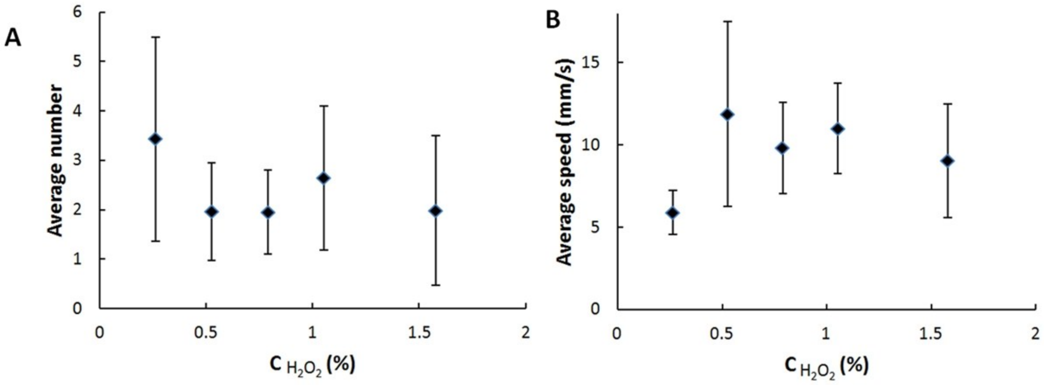

Fig.S3. (A) Average number of Au-GCB-Pt moved in each frame as a function of H_2O_2 concentration. (B) Average speed of Au-GCB-Pt in fuel solution. The concentration of SDS was 0.021 g/L. The concentration of Na_2SO_4 was 0.185 M.

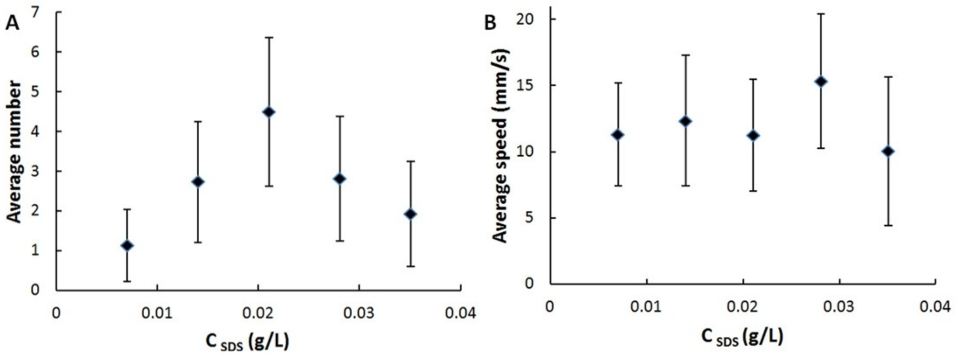

Fig.S4. (A) average number of Au-GCB-Pt moved in each frame as a function of SDS concentration. (B) Average speed of Au-GCB-Pt in fuel solution. The concentration of H_2O_2 was 0.526%. The concentration of Na_2SO_4 was 0.185 M.

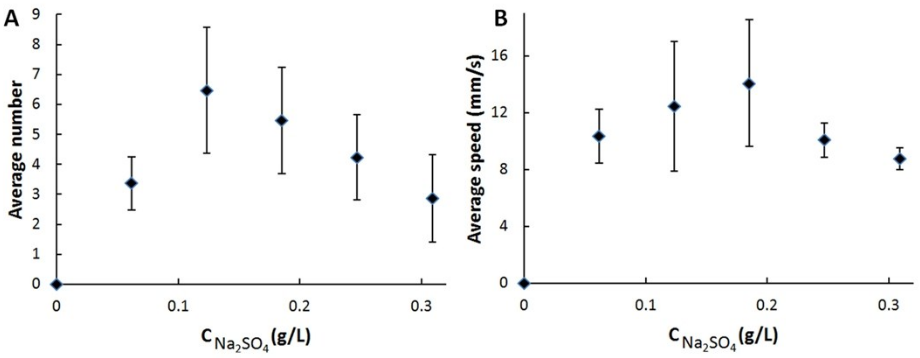

Fig.S5. (A) average number of Au-GCB-Pt moved in each frame as a function of Na_2SO_4 concentration. (B) Average speed of Au-GCB-Pt in fuel solution. The concentration of H_2O_2 was 0.526%. The concentration of SDS was 0.021 g/L.

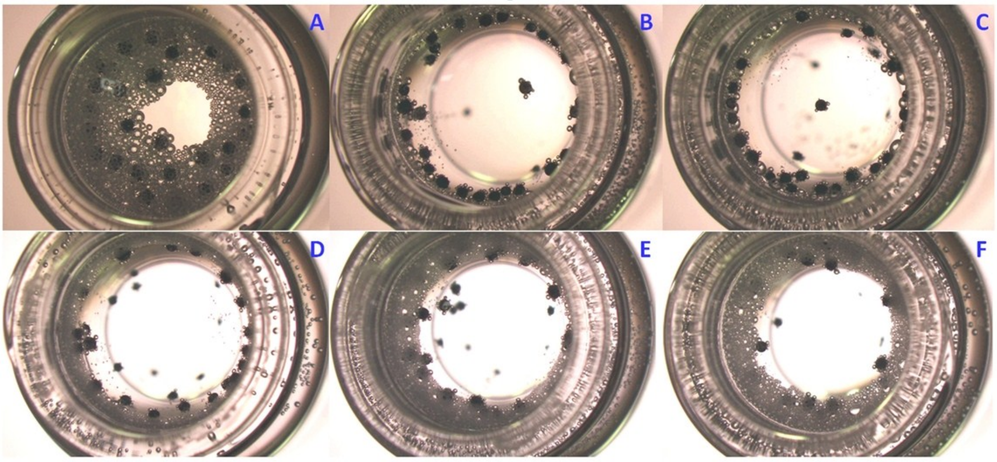

Fig.S6. Top view of Au-GCB-Pt in fuel solution. The concentration of H_2O_2 was 0.526%. The concentration of SDS was 0.021 g/L. The concentration of Na_2SO_4 from A to F was 0, 0.062, 0.123, 0.185, 0.247, and 0.308 M, respectively.

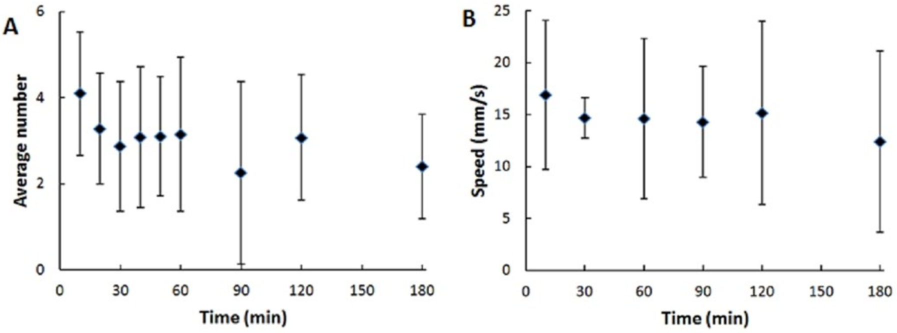

Fig.S7. (A) Average number of Au-GCB-Pt moved in each frame and (B) Average speed of Au-GCB-Pt in fuel solution containing 0.526% H_2O_2, 0.021 g/L SDS and 0.185 M Na_2SO_4.
